# A Review of Risk Factors and Timing for Postoperative Hematoma After Thyroidectomy: Is Outpatient Thyroidectomy Really Safe?

**DOI:** 10.1007/s00268-012-1682-1

**Published:** 2012-06-20

**Authors:** Brian Hung-Hin Lang, Patricia Chun-Ling Yih, Chung-Yau Lo

**Affiliations:** 1 Department of Surgery, The University of Hong Kong, Hong Kong SAR, China; 2 Division of Endocrine Surgery, Department of Surgery, Queen Mary Hospital, 102 Pokfulam Road, Hong Kong SAR, China

## Abstract

**Background:**

Although postoperative hematoma after thyroidectomy is uncommon, patients traditionally have been advised to stay overnight in the hospital for monitoring. With the growing demand for outpatient thyroidectomy, we assessed its safety and feasibility by evaluating the potential risk factors and timing of postoperative hematoma after thyroidectomy.

**Methods:**

From 1995–2011, 3,086 consecutive patients underwent thyroidectomy at our institution; of these, 22 (0.7 %) developed a postoperative hematoma that required surgical reexploration (group I). Potential risk factors were compared between group I and those without hematoma (*n* = 3,045) or with hematoma but not requiring reexploration (*n* = 19; group II). Variables that were significant in the univariate analysis were entered into multivariate analysis by binary logistic regression analysis.

**Results:**

Group I was significantly more likely to have undergone previous thyroid operation than group II (27.3 vs. 8.2 %, *p* = 0.007). The median weight of excised thyroid gland (71.8 vs. 40 g, *p* = 0.018) and the median size of the dominant nodule (4.1 vs. 3 cm, *p* = 0.004) were significantly greater in group I than group II. Previous thyroid operation (odds ratio (OR) = 4.084; 95 % confidence interval (CI), 1.105–15.098; *p* = 0.035) and size of dominant nodule (OR = 1.315; 95 % CI, 1.024–1.687; *p* = 0.032) were independent factors for hematoma. Sixteen (72.7 %) had hematoma within 6 h, whereas the other 6 (27.3 %) had hematoma at 6–24 h.

**Conclusions:**

Previous thyroid operation and large dominant nodule were independent risk factors for hematoma requiring surgical reexploration. Given that a quarter of hematoma occurred between 6 to 24 h after surgery, routine outpatient thyroidectomy could not be recommended.

## Introduction

Thyroidectomy is not only one of the most commonly performed surgical procedures but also one of the safest in experienced hands [1]. The common surgical indications include multinodular goiter with compressive symptoms, suspicious or indeterminate nodule, proven malignancy, relapsed Grave’s disease, and retrosternal goiter [2, 3]. Although a great deal of work has been published focusing on the prevention and reduction of thyroidectomy-related complications, such as recurrent laryngeal nerve injury and postoperative hypoparathyroidism in the literature [4–6], relatively fewer studies have focused on prevention and risk factors of postoperative hemorrhage or hematoma after thyroidectomy [7–10]. This might be because with improved surgical technique and meticulous hemostasis, clinically significant hematoma occurs uncommonly and even when it does, if managed timely and appropriately, patients are expected to fully recover afterwards. Nevertheless, it should be reminded that it is a potentially life-threatening complication and in a recent territory-wide audit of 1,616 patients in 17 local hospitals, two deaths resulted from thyroidectomy with one directly related to postoperative hematoma [11]. Furthermore, it would be timely to refocus on this particular complication more critically, because there has been a gradual shift of practice from inpatient to outpatient or same-day thyroidectomy [12–14]. Despite the ongoing debate on the safety of outpatient thyroidectomy, there is evidence to suggest that outpatient thyroidectomy is becoming increasingly widespread particularly in the United States [12, 13]. Although we fully acknowledge the fact that hematoma formation is only one of many issues to consider in the assessment of outpatient thyroidectomy, nevertheless it is the most life-threatening and deserves more critical evaluation. With this background, the present study was designed to assess the safety and feasibility of outpatient thyroidectomy by evaluating the potential risk factors and timing of postoperative hematoma in a predominantly inpatient thyroidectomy cohort.

## Patients and methods

From 1995 to 2011, 3,086 consecutive patients who underwent thyroidectomy in our institution were reviewed retrospectively. All were operated on by two endocrine surgeons (BHL, CYL) and managed afterwards by the same surgical team. With the exception of six (0.2 %) patients who were discharged on the same day, all postoperative patients were admitted overnight and monitored hourly for the first 12–18 h. The median hospital duration was 2 (range 1–6) days. During the study period, 22 (0.7 %) patients were identified to have clinically significant postoperative hemorrhage or hematoma after thyroidectomy (i.e., those requiring surgical reexploration; group I). One particular patient required two surgical reexplorations 24 h apart. For this study, only the first reexploration was recorded. An additional 19 patients developed hematoma but did not require surgical reexploration, because their hematoma was only discovered some days after operation and was asymptomatic. These 19 patients were grouped into the nonhematoma group, and so the total number of patients in this group was 3,064 (*n* = 3,045 + 19; group II). All relevant clinical, laboratory, radiologic, and perioperative data were collected prospectively and follow-up data were regularly updated in a computerized database. To evaluate the potential risk factors for postoperative hematoma formation, preoperative and operative factors were compared between groups I and II. For the calculation of the onset of postoperative hematoma, it was taken as the time interval or difference from the end of the procedure to the time when the hematoma was first found by a medical or nursing staff or noticed by the patient, whichever came first. The present study protocol was approved by the local institutional review board.

### Surgery and postoperative care

Surgical techniques, postoperative care, and follow-up protocol were similar throughout the study period and have been previously described [15]. Preoperative patients were instructed to stop antiplatelet agents at least 5 days before the operation and to resume it on the following day. For those taking warfarin, patients were asked to stop 4 days before operation and to start low molecular weight heparin (LMWH) at a therapeutic dose. LMWH was stopped 8–10 h before the operation. Thyroidectomy was performed in a standardized manner under general anesthesia. The strap muscles were separated in the midline and retracted laterally. Both recurrent laryngeal nerves and parathyroid glands were routinely identified and preserved. Any devascularized parathyroid glands were immediately minced and autoimplanted to the ipsilateral sternocleidomastoid muscle. Meticulous hemostasis was performed before skin closure. Suction drain was placed selectively, mostly for large-sized retrosternal goiters or those requiring concomitant neck dissections. Since 2008, some of the cases were performed endoscopically by the transaxillary route (*n* = 80) or by the minimally invasive video-assisted approach (MIVAT; *n* = 40) [16, 17]. The rest were performed through the standard Kocher incision. Also since 2007, an alternate energy source, such as the Harmonic scalpel focus (Ethicon, Cincinnati, OH) or Sonosurg (Olympus, Japan), was used selectively for hemostasis and cutting.

### Statistical analysis

Statistical analysis was performed by χ^2^ or Fisher’s exact test to compare categorical variables, and Mann–Whitney *U* test was used to compare continuous variables between the two groups. Continuous variables were expressed as medians with ranges. Variables that were significant in the univariate analysis were entered into multivariate analysis. Binary logistic regression analysis with a variable entrance criterion of 0.05 or less was conducted to identify independent risk factors for hematoma requiring surgical reexploration. All statistical analyses were performed by using SPSS^®^ version 18.0 (SPSS, Inc., Chicago, IL).

## Results

Table 1 shows a comparison of patient baseline characteristics and operative findings between groups I and II. Baseline patient characteristics, such as age, sex, and clinical presentation, were comparable between the two groups. The final pathology also was comparable between the two groups with nodular hyperplasia or multinodular goiter accounting for almost half of all pathologies. The proportion of patients taking an antiplatelet agent or warfarin before the operation was similar between the two groups. Although the proportion of patients who received radioiodine treatment was similar, significantly more patients in group I had previous thyroid operations than patients in group II (27.3 vs. 8.2 %, *p* = 0.007). When the study period was divided into three time periods (I: 1995–2000; II: 2001–2006; III: 2007–2011) and they were compared between the two groups, there was no significant difference in the rate of hematoma requiring surgical reexploration. Hematomas that required surgical reexploration during the time periods I, II, and III were 6 of 751 (0.8 %), 8 of 1,167 (0.7 %), and 8 of 1,168 (0.7 %), respectively. In terms of operative findings, the extent of thyroidectomy (hemithyroidectomy/total or near-total thyroidectomy), surgical approach (open/endoscopic), hemostatic technique (clamp and tie/alternate energy source), total operating time, and drain placement were comparable between the two groups. However, group I had significantly heavier excised thyroid gland (71.8 vs. 40 g, *p* = 0.018) and larger-sized dominant nodule (4.1 vs. 3.0 cm, *p* = 0.004) than group II.

**Table 1:** Comparison of patient demographics and operative findings between those requiring exploration of hematoma (group I) and those either with no hematoma or not requiring exploration of hematoma (group II)

	Group I (*n* = 22)	Group II (*n* = 3,064)	*p* Value
Age at operation (years)	52.5 (14.4–74)	48 (6–91.1)	0.34
Sex			0.161
Male	7 (31.8)	562 (18.3)	
Female	15 (68.2)	2502 (81.7)	
Presented with pressure symptoms	8 (36.4)	782 (25.5)	0.213
Primary pathology			
Nodular hyperplasia/MNG	10 (45.5)	1324 (43.2)	0.217
Grave’s disease	4 (18.2)	411 (13.4)	0.525
Malignancy	3 (13.6)	551 (18)	1
Miscellaneous	5 (22.7)	778 (25.4)	0.512
On an antiplatelet agent	1 (4.5)	107 (3.5)	0.545
On warfarin	0 (0)	15 (0.5)	1
Previous thyroid operation	6 (27.3)	251 (8.2)	**0.007**
Previous radioiodine treatment	0 (0)	22 (0.7)	1
Period of operation			0.949
1995–2000	6 (27.3)	745 (24.3)	
2001–2006	8 (36.4)	1159 (37.8)	
2007–2011	8 (36.4)	1160 (37.9)	
Extent of thyroidectomy			0.81
Hemithyroidectomy	8 (36.4)	968 (31.6)	
Total or near-total thyroidectomy	14 (63.6)	2096 (68.4)	
Surgical approach			0.646
Open	22 (100)	2947 (96.2)	
Endoscopic	0 (0)	117 (3.8)	
Hemostatic technique			1
Clamp and tie	21 (95.5)	2891 (94.4)	
Alternate energy source	1 (4.5)	173 (5.6)	
Total operating time (min)	120 (45–190)	113 (35–420)	0.843
Weight of excised gland (g)	71.8 (9–152)	40 (1.8–727)	**0.018**
Size of dominant nodule (cm)	4.1 (1.2–8.3)	3 (0.5–15)	**0.004**
Drain placement	7 (31.8)	828 (27)	0.47

Continuous variables are expressed as median (range); categorical variables are expressed as number (percentage)

*MNG* multinodular goiter

Bold signifies *p*-value < 0.05

Table 2 shows a multivariable analysis of potential risk factors for hematoma requiring exploration. Variables that were significant in the univariate analysis were entered into the multivariate analysis. History of previous thyroid operation (odds ratio (OR) = 4.084; 95 % confidence interval (CI), 1.105–15.098; *p* = 0.035) and size of dominant nodule (OR = 1.315; 95 % CI, 1.024–1.687; *p* = 0.032) were the independent factors for hematoma formation requiring surgical reexploration. Because weight of excised gland and size of dominant nodule were somewhat interrelated, each of these covariates was entered separately with history of previous thyroid resection, but the final results were similar to when all three covariates were entered together. When the covariate, size of dominant nodule, was converted into a binary variable using 3 cm (the median) as the cutoff and entered into the model, relative to size ≤3 cm, size of dominant nodule >3 cm had an OR of 4.54 (95 % CI, 1.22–16.92; *p* = 0.024).

**Table 2:** Multivariable analysis of risk factors for hematoma requiring exploration (*n* = 22)

Covariates	β-Coefficient	Odds ratio (95 % confidence interval)	*p* Value
Previous thyroid operation			
No		1	
Yes	1.407	4.084 (1.105–15.098)	**0.035**
Weight of excised gland (g)	0.001	1 (0.992–1.009)	0.911
Size of dominant nodule (cm)^a^	0.273	1.315 (1.024–1.687)	**0.032**

^a^When entered as a categorical variable with 3 cm being the cutoff, the odds ratio for size of dominant nodule >3 cm became 4.537 (95 % CI, 1.217–16.917; *p* = 0.024) and the odds ratio for previous thyroid operation became 3.835 (95 % CI, 1.034–14.216; *p* = 0.044)

Bold signifies *p*-value < 0.05

**Fig. 1:**
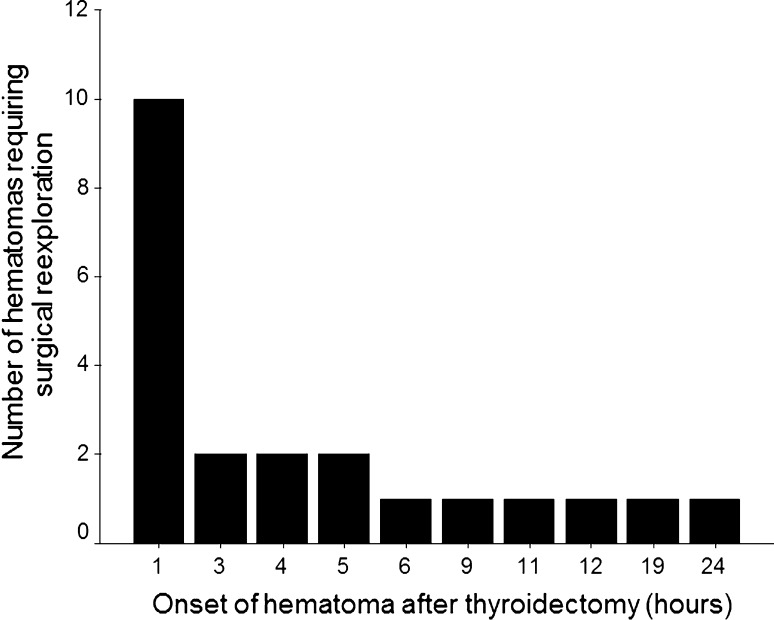
Distribution of time interval from initial operation to onset of hematoma of the 22 patients requiring surgical reexploration

Figure 1 shows the distribution of time interval from end of operation to the onset of hematoma. The median onset of hematoma was 3 h (range <1 to 24) hours. Ten of the 22 (45.5 %) patients were found to have hematoma at the recovery room. When patients were categorized according to the specific time intervals (<6, 6–24, and >24 h), 16 of 22 (72.7 %) had hematoma within 6 h, whereas the other 6 (27.3 %) had hematoma between 6 to 24 h. No patient developed hematoma requiring reexploration after 24 h.

Risk factors for nonoperative hematoma (i.e., not requiring reexploration) also were determined by comparing patient baseline characteristics and operative findings between those who had no hematoma (*n* = 3,045) and those who had nonoperative hematoma (*n* = 19). Male sex was the only significant factor for predicting nonoperative hematoma with OR = 3.27 (95 % CI, 1.31–8.2; *p* = 0.011). Factors, such as history of previous thyroid resection, weight of excised gland, and size of dominant nodule were not found to be significant.

## Discussion

Thyroidectomy remains one of the most commonly performed surgical procedures with a low morbidity rate in experienced hands [1, 11]. Hematoma formation after thyroidectomy is a well-known complication, but with improved surgical technique and meticulous hemostasis, it has become a rare occurrence [13]. Nevertheless, if not detected early and managed properly, it is potentially life-threatening. In a recent territory-wide audit of 17 local hospitals, at least one postoperative death was attributed to postoperative hemorrhage [11]. In our series, the overall rate of hematoma was 0.7 %; this rate appeared to be relatively unchanged throughout the study period (*p* = 0.949). This rate also appeared to be consistent with most other single-institution series but lower than many multicenter or nationwide series [7–10, 13, 18–22]. This could be attributed to the fact that all the operations were performed by two endocrine surgeons whose surgical practice predominantly involved thyroidectomy. Given the possible correlation between rate of hematoma and case volume, perhaps a slightly higher rate of hematoma would be expected in less specialized units [21].

One of the main purposes of conducting this study was to evaluate whether outpatient thyroidectomy was safe and feasible in our setting, because in our experience, the possibility of hematoma formation remains one of the major reasons for keeping patients overnight after surgery. Although there are other conditions, such as postoperative emesis, acute retention of urine, and symptomatic hypocalcemia, which may occur in early postoperative period and may favor inpatient instead of outpatient procedure, most of these are not life-threatening and could be greatly minimized with early administration of medication and supplementation [13, 23]. It was stimulating to see that in one of largest outpatient thyroidectomy series, up to 86 % of all cases were performed in an outpatient setting [13]. In the era of cost containment, it is expected that this would continue and become more widespread across the United States [12, 13, 23]. According to our own territory-wide data, outpatient thyroidectomy only accounted for 3.5 % of all cases and so potentially there are still many patients eligible for outpatient thyroidectomy [11]. Given this background, our study was divided into two parts. The first part was to determine risk factors for clinically significant hematoma. To our knowledge, although most studies could not identify any clear risk factors for hematoma, a few studies reported that older age, male gender, thyroid malignancy, and greater extent of surgery as possible risk factors [7–10, 18–22]. However, none of four factors were significant in our univariate and multivariate analyses. Instead, history of previous thyroid operation and size of dominant nodule were identified as independent risk factors for hematoma. For the latter factor, those with size of dominant nodule >3 cm had an OR of 4.54 (95 % CI, 1.22–16.92; *p* = 0.024) or approximately 4.5 times higher risk than that of size ≤3 cm. We postulate that the larger size nodule tended to result in larger dissection and raw surfaces, which might have led to increased rate of hematoma. However, despite this postulation, it was interesting to note that the hematoma rate between hemithyroidectomy and total thyroidectomy were not different (*p* = 0.81). The alternative explanation might be related to the relatively large volume of potential dead space, which tends to encourage hematoma formation. Although selective placement of suction drain may theoretically reduce the volume of dead space and hematoma, our data did not support this [24]. Regarding previous thyroid operation as risk factor, we postulate that perhaps due to the greater amount of scar tissue present in the reoperated field, it would have been more difficult for the dead space after thyroidectomy to collapse by itself and for smaller vessels to vasoconstrict and these might contribute to the risk of hematoma. However, it was interesting to note that none of these factors were risk factors for the nonoperative hematoma group. In fact, male sex was the only risk factor. During the study period, these 19 patients with nonoperative hematoma were simply managed expectantly and followed up weekly in our clinic until the hematoma resolved. In our experience, their hematoma spontaneously resolved during a period of 4–6 weeks.

The second part of our analysis focused on the timing or onset of hematoma. Similar to previous studies, we found that the majority (72.7 %) of hematomas requiring reexploration occurred within the first 6 h after thyroidectomy [25]. In this series, the median onset of hematoma was 3 (range <1 to 24) hours with ten (45.5 %) patients noted with hematoma in the recovery room. Six hours was used as the cutoff because in the outpatient setting, patients would be monitored for approximately 6 h before discharge from the hospital [7, 8]. Therefore, hypothetically, most, if not all, of these hematomas would be detected and managed appropriately in the day-surgery clinic. However, the most concerning issue was that hematomas of six (27.3 %) patients were detected after 6–24 h. If these had been performed as outpatient procedures, the patients would have been discharged from the hospital. In our opinion, although this select group may only constitute 6 of 3,086 or 0.19 % of the whole cohort, if their hematoma was not managed appropriately outside the hospital setting, their hematoma would cause a potentially life-threatening airway compromise or obstruction and possibly lead to a preventable death. In fact, based on a similar conception, Schwartz et al. calculated that 94 hematoma-related deaths per 100,000 thyroidectomies could be prevented if patients are monitored for 24 h instead of 6 h. If a similar calculation was performed for this study, the number of preventable hematoma-related deaths for 24-h monitoring as opposed to 6-h monitoring would have been 194. Although a selective approach to outpatient thyroidectomy based on risk factors (e.g., those with no previous thyroid operation and/or with ≤3 cm dominant nodule) might seem realistically possible, in our opinion, there was still going to be a very small possibility that providing less than 24-h postoperative monitoring could lead to a preventable hematoma-related death.

Despite our findings, the total number of hematomas was relatively small, which limited the power of our study to identify smaller effects. Therefore, we could not rule out other possible risk factors for hematoma. Also, the present analysis was potentially subjected to a degree of institution and referral biases, because our center is an academic tertiary care institute to which more complex cases often are referred from other hospitals. A future, multicenter study involving other institutions in our territory would be desirable to further confirm our findings.

## Conclusions

Our incidence of clinically significant hematoma formation after thyroidectomy was 0.7 %, and this rate remained relatively unchanged throughout the past 16 years. History of previous thyroid operation and large (>3 cm) dominant nodule were identified as independent risk factors for hematoma formation after thyroidectomy. The majority (72.7 %) of hematomas developed within 6 h after thyroidectomy, whereas the rest (27.3 %) developed between 6 to 24 h. Given the fact that some of these hematomas may occur up to 24 h after surgery, routine outpatient or same-day thyroidectomy could not be recommended in our setting.
